# Diversity in Carbapenemases in Enterobacterales in Southeastern Austria Before and During the COVID-19 Pandemic

**DOI:** 10.3390/pathogens14111130

**Published:** 2025-11-06

**Authors:** Andrea Grisold, Lena Gruber, Yasmin Mandl, Josefa Luxner, Branka Bedenić, Gernot Zarfel

**Affiliations:** 1 Diagnostic and Research Center for Molecular BioMedicine, Medical University Graz, 8010 Graz, Austria; andrea.grisold@medunigraz.at (A.G.); josefa.luxner@medunigraz.at (J.L.); 2 Biomedical Science, University of Applied Sciences, 8020 Graz, Austria; 3 Biomedical Research Center Šalata-BIMIS, Department for Clinical Microbiology and Infection Prevention and Control, University of Zagreb School of Medicine, 10000 Zagreb, Croatia; branka.bedenic@kbc-zagreb.hr

**Keywords:** carbapenem-resistant Enterobacterales, *Klebsiella pneumoniae*, *Enterobacter cloacae*, metallo-ß-lactamase, VIM-1, OxA-48-like, multi-resistant-bacteria

## Abstract

The COVID-19 pandemic has profoundly influenced healthcare systems and infection control worldwide, with important implications for the epidemiology of antimicrobial resistance. This study examined the prevalence and characteristics of carbapenem-resistant Enterobacterales (CRE) isolates in Southeastern Austria from 2018 to 2022 to assess potential pandemic-related effects. A total of 63 isolates were analyzed using phenotypic and molecular methods, including carbapenemase detection, genotyping, and multilocus sequence typing. The number of CRE isolates appeared to decline during the pandemic years (2021–2022) compared to the pre-pandemic period, with *Enterobacter cloacae* notably detected in both full pandemic years. Carbapenemase-producing CRE accounted for 44 out of the 63 isolates (69.8%), with metallo-beta-lactamases (VIM-1 and NDM-1) and OXA-48-like carbapenemases predominating. Resistance mechanisms not based on carbapenemase production were more common before the pandemic but rarely detected thereafter. To our knowledge, this is the first report of dual-carbapenemase-producing CRE isolates in Austria. Multi-locus-sequence typing indicated limited nosocomial transmission, with most isolates representing independent introductions linked to external sources. The decline in CRE prevalence may reflect reduced international travel and healthcare access during the pandemic, which could have limited the importation of resistant strains. These findings reflect the potential role of global mobility in the spread of CRE and illustrate how public health interventions can shape antimicrobial resistance trends.

## 1. Introduction

Carbapenem-resistant Enterobacterales (CRE) are among the most concerning antibiotic-resistant pathogens, as infections caused by these organisms often leave only limited therapeutic options. In addition to carbapenem resistance, CRE often display co-resistance to fluoroquinolones, folate antagonists, and, to a lesser extent, aminoglycosides, further limiting treatment options [1,2,3].

A central mechanism of carbapenem resistance is the production of carbapenemases, with several enzyme families exhibiting carbapenemase activity. Among the Ambler class A serine hydrolases, KPC and certain members of the GES family, notably GES-5 and its variants, are commonly found in CRE. In the past, metallo-beta-lactamases (MBLs, Ambler class B) from the NDM, VIM, and IMP families were the most prevalent enzymes associated with carbapenem resistance in these bacteria. In the last decade, OXA-48-like carbapenemases (class D) have expanded rapidly across Europe, surpassing other families in prevalence [1,4,5,6].

There are several very successful carbapenem resistant high-risk clones among *Klebsiella* (*K.*) *pneumoniae*. One of the first was ST258, carrying blaKPC-2 and blaKPC-3 genes which originated from the USA (North Carolina) and spread later globally. In Italy, this clone was recently associated with colistin resistance due to mutations of the *mgrB* gene. The therapeutic options in such strains are very limited and the infections are associated with a high mortality rate. Later, other successful clones were reported, for example ST11 and ST340 spreading blaNDM-1 and blaNDM-5 genes [7,8,9].

In Austria, awareness of CRE has increased over the past decade, accompanied by reports of all major species and carbapenemase families, including NDM, KPC, and OXA-48-like enzymes. Notably, GES-type carbapenemases have not been identified, distinguishing Austria’s resistance profile from other European regions. Despite these developments, CRE prevalence remains lower than in neighboring countries [10,11,12].

The COVID-19 pandemic disrupted healthcare systems and altered human contact patterns worldwide, factors that could influence the spread of CRE. However, studies assessing the pandemic’s impact on resistant bacteria have yielded inconsistent results, with no clear consensus emerging on whether CRE prevalence has increased or declined [13,14,15].

The present study investigated CRE isolates from Southeastern Austria collected between 2018 and 2022. Antimicrobial resistance profiles and carbapenemase genes were characterized by phenotypic and molecular methods, including a novel chip-based assay. By comparing pre-pandemic and pandemic years, this work provides insights into temporal shifts in CRE prevalence and mechanisms of resistance. The findings underscore the role of international mobility and healthcare access in shaping resistance trends and emphasize how public health interventions may influence the dissemination of CRE.

## 2. Materials and Methods

### 2.1. Bacterial Isolates

The study was conducted at the Institute of Hygiene, Microbiology and Environmental Medicine, Medical University of Graz, Austria. Clinical samples were obtained from the University Hospital of Graz (approximately 1200 beds), eight peripheral hospitals, and local practitioners throughout the state of Styria in Southeastern Austria. Between 2018 and 2022, the annual hospital occupancy remained consistent, with total patient numbers ranging from 10,731 to 11,666 per year (10,969 in 2018; 11,353 in 2019; 10,731 in 2020; 11,666 in 2021; and 11,638 in 2022). The total number of microbiological samples analyzed exhibited only minor year-to-year variation, increasing from 50,174 in 2018 to 54,470 in 2022 (50,933 in 2019; 51,159 in 2020; and 54,181 in 2021). IPC programs for CRE screening were not modified during the observation period, which was reflected in the stable number of screening samples ranging from 2525 to 3753 per year (2710 in 2018; 3753 in 2019; 2736 in 2020; 2525 in 2021; and 2532 in 2022).

To calculate the percentage of CRE among all Enterobacterales, the number of patients with confirmed Enterobacterales isolates was determined. Among hospitalized patients, the annual numbers were as follows: 4380 in 2018, 4429 in 2019, 3572 in 2020, 3707 in 2021, and 3905 in 2022. In the outpatient setting, the corresponding numbers were 12,914 in 2018, 12,299 in 2019, 12,073 in 2020, 12,447 in 2021, and 13,238 in 2022.

Bacterial identification and antibiotic susceptibility testing were performed using the semi-automated VITEK II instrument (bioMérieux, Marcy l’Etoile, France). All primary CRE isolates were routinely stored at −70 °C.

### 2.2. Antibiotic Susceptibility Test

The isolates were characterized for their resistance pattern to 11 antibiotics by agar diffusion susceptibility testing according to the European Committee on Antimicrobial Susceptibility Testing (EUCAST) with piperacillin/tazobactam 30.0 μg/6.0 μg, cefotaxime 5.0 μg, ceftazidime 10.0 μg, ceftazidime/avibactam 10.0 μg/4.0 μg, cefepime 30.0 μg, aztreonam 30.0 μg, imipenem 10.0 μg, meropenem 10.0 μg, ertapenem 10.0 μg, cefiderocol 30.0 μg, gentamicin 10.0 μg, trimethoprim/sulfamethoxazole 1.25 μg/23.75 μg, ciprofloxacin 5.0 μg and moxifloxacin 5.0 μg (all with BD BBLTM Sensi-DiscTM paper disks; BD, Sparks, MD, USA). *Escherichia* (*E.*) *coli* ATCC 25922, *K. pneumoniae* 700603 and *Pseudomoas* (*P.*) *aeruginosa* ATCC 27853 were used as reference strains. The inhibition zone diameters were interpreted according to EUCAST guidelines. There are limited breakpoints for moxifloxacin, breakpoint for resistance is >0.25 mg/L or inhibition zone size < 22 mm (EUCAST version 14.0, Clinical Breakpoint Tables, valid from 1 January 2024). Resistance breakpoints for cefiderocol are >2 mg/L or <23 mm for disk-diffusion test. For colistin susceptibility testing *E. coli* NCTC 13846 (mcr-1 positive) was used, resistance breakpoint is >2 mg/L. Colistin susceptibility testing was performed via broth microdilution using the UMIC Colistine Test Kit (Bruker, Daltonics, Bremen, Germany) according to EUCAST because Colistin does not penetrate into agar.

Reference strains were included in each batch of testing, and results for all antimicrobial susceptibility tests were within the acceptable EUCAST ranges, confirming assay performance throughout the study period.

### 2.3. Carba NP Test

The RAPIDEC^®^ CARBA NP Test from bioMérieux was used to determine whether the isolates expressed carbapenemase enzymes. This method reliably differentiates carbapenemase-producing isolates from non-producers

### 2.4. Genotyping by Inter-Array Kit CarbaResist

The isolates were genotyped using an inter-array chip according to the manufacturer’s recommendations (Inter-array fzmb GmbH, Bad Langensalza, Germany). The CarbaResist inter-array genotyping kit (Bad Langensalza, Germany) allows DNA-based detection of the most common β-lactamases and other resistance genes in multidrug-resistant Gram-negative bacteria from bacterial cultures.

After isolating RNA-free, unfragmented genomic DNA from pure material, the DNA was amplified and internally labeled with biotin-dUDP using a linear PCR amplification protocol, employing only the antisense primer of the different targets. The resulting products were single-stranded DNA (ssDNA). This biotin-labeled ssDNA was then transferred into an ArrayWell and hybridized to DNA oligonucleotide microarrays with 230 probes for different carbapenemases, *bla*_ESBL_ and *bla*_ampC_ genes, as well as other relevant antibiotic resistance genes.

After hybridization and subsequent washing, HRP-conjugated streptavidin bound to the hybridized biotin-labeled ssDNA strains and visualized them in an enzymatic reaction. The evaluation of the spots and their intensities was performed automatically using a digital image of the microarray with an INTER-VISION Reader (V1.1.8). The samples were automatically analyzed for the presence or absence of specific probes, cross-checked against a database, and information on existing resistances and possible bacterial species was provided.

### 2.5. Molecular Detection of Resistance Genes

The same template DNA used for genotyping with the Inter-Array Kit CarbaResist was utilized. Genes encoding resistance to carbapenems, including class A (*bla*_KPC_, *bla*_GES_), class B (*bla*_VIM_, *bla*_IMP_, and *bla*_NDM_), and class D carbapenemases or carbapenem-hydrolyzing oxacillinases (bla_OXA-48-like_), were amplified and sequenced as previously described [16,17,18]. Control strains were isolates of *E. coli* (*bla*_NDM-1_), *K. pneumoniae* (*bla*_NDM-1_), *K. pneumoniae* (*bla*_VIM-1_), *Klebsiella* (*K*.) *oxytoca* (*bla*_GES-1_) and *P. aeruginosa* (*bla_I_*_MP-1_), all confirmed by sequencing.

### 2.6. Multilocus Sequence Typing (MLST)

MLST for *E. coli* was performed according to the MLST Databases at UoW (The University of Warwick) (https://enterobase.warwick.ac.uk/species/ecoli/allele_st_search). Database access for sequence comparison: accessed on 1 February 2023.

MLST for *K. pneumoniae* was performed according to Institute Pasteur MLST (https://bigsdb.pasteur.fr/klebsiella/). Database access for sequence comparison: accessed on 30 January 2023.

MLST for *K. oxytoca* was performed according to the *K. oxytoca* MLST website (http://pubmlst.org/koxytoca/) sited at the University of Oxford. Database access for sequence comparison: accessed on 1 February 2023.

MLST for *Enterobacter* (*E.*) *cloacae* was performed according to the *E. cloacae* MLST website (http://pubmlst.org/ecloacae/). Database access for sequence comparison: accessed on 5 December 2023.

MLST for *Citrobacter* spp. was performed according to the *Citrobacter* spp. MLST website (https://pubmlst.org/organisms/citrobacter-spp). Database access for sequence comparison: accessed on 6 December 2023.

### 2.7. Statistical Analyses

Year-to-year comparisons of CRE prevalence among hospitalized patients were per-formed using Fisher’s exact test. Effect sizes were expressed as relative risks (RR) with corresponding 95% confidence intervals (CI).

Proportions for resistance to tested antibiotics were calculated for each year, and 95% confidence intervals were determined using the Wilson method for binomial data.

## 3. Results

A total of 63 single-patient, non-duplicate CRE isolates were included in this study. Of these, 20 were collected in 2018, 17 in 2019, 12 in 2020, five in 2021, and nine in 2022. Only the first isolate from each patient was considered, regardless of whether it originated from a clinical or screening sample. The majority of isolates (n = 56) originated from hospitalized patients, while only seven were obtained from outpatients (one each in 2019 and 2021, two in 2018, three in 2020 and none in 2022). The isolates comprised 20 *K. pneumoniae*, 11 *E. cloacae* complex, 10 *Klebsiella* (*K.*) *aerogenes*, six *E. coli*, six *Serratia* (*S.*) *marcescens*, five *K. oxytoca*, two *Citrobacter* (*C.*) *freundii*, and one isolate each of *Proteus* (*P.*) *mirabilis*, *Proteus* (*P.*) *vulgaris*, and *Raoultella* (*R.*) *ornithinolytica*. (Table 1).

When adjusted for the total number of patients, analysis was restricted to hospitalized patients due to the low number of CRE cases in the outpatient setting. Among hospitalized patients, the proportion of carbapenem-resistant isolates showed a consistent decline over the five-year study period, reaching its lowest value of 0.11% in 2021 and decreasing overall from 0.41% in 2018 to 0.23% in 2022.

Although the overall trend (hospitalized patients) indicates a decrease in CRE preva-lence, year-to-year comparisons did not consistently yield statistically significant differ-ences, which is at least partly attributable to the relatively small number of isolates avail-able per year. The most pronounced decrease in hospitalized patients was observed between 2020 and 2021; however, this comparison did not remain statistically significant (Fisher’s exact test, *p* = 0.091, RR = 2.49, 95% CI 0.88–7.06). Nevertheless, it is noteworthy that in 2021—the first year fully encompassed by the COVID-19 pandemic—statistically significant reductions in prevalence were observed compared with the pre-pandemic years 2018 (*p* = 0.009, RR = 3.39, 95% CI 1.27–9.01) and 2019 (*p* = 0.033, RR = 2.85, 95% CI 1.05–7.71). Taken together, the data suggest a declining trend in CRE prevalence during the observation period, but the findings must be interpreted with caution given the limited number of isolates and the potential impact of random variation.

For the remainder of the manuscript, inpatient and outpatient isolates were pooled for analysis. This was necessary due to the limited number of outpatient samples, which precluded robust statistical comparison between the two groups.

Analysis of individual bacterial species revealed shifts in their prevalence. *E. cloacae*, previously among the three most frequently identified species, was entirely absent during the two full years of the pandemic. A similar pattern was observed for *K. aerogenes*, which began to decline noticeably in 2020 and was no longer detected in 2021.

### 3.1. Carbapenemases

**Figure 1:**
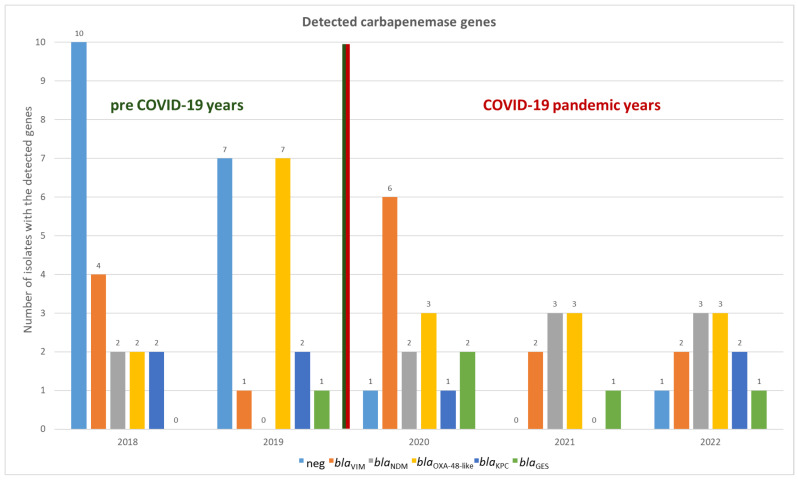
Detected carbapenemases (grouped by carbapenemase protein families) from 2018 to 2022 in isolated carbapenem-resistant Enterobacterales. “neg” refers to 19 isolates that did not show a positive signal for any of the tested genes. Isolates with two or three different carbapenemase genes are not listed separately; instead, the genes are categorized under their respective families. In six isolates, two genes were detected in each, resulting in a total of 55 genes listed, which were found in the 44 carbapenemase-positive isolates. Green-red dividing line between 2019 and 2020, representing the graphic separation of the pre-COVID years from the COVID-19 pandemic years.

A total of 55 carbapenemase genes were identified in 44 out of 63 isolates, corresponding to 69.8% of the total sample. The only discrepancy between conventional PCR and CarbaResist kit was observed in *S. marcescens*: for five out of six isolates, the chip analysis suggested the presence of a GES-type carbapenemase. All isolates yielded positive *GES* signals on the CarbaResist inter-array chip, with signal intensities exceeding 0.9, comparable to the positive control. However, subsequent PCR testing did not confirm the presence of *bla*_GES_ genes in any isolate (see Supplementary Figure S1). This discrepancy indicates that the chip results should be interpreted with caution, as unspecific hybridization or probe cross-reactivity may have contributed to false-positive signals. Consequently, all *bla*_GES-_positive chip results were reported as positive according to chip, not confirmed by PCR. In all other cases, the results of the microarray analysis were consistent; no carbapenemase gene was detected by only one method (Figure 1 and Supplementary Table S1).

No carbapenemase genes were detected in the remaining 19 isolates (30.2%). Of these 19 isolates, seven were found to harbor an efflux pump as the likely resistance mechanism, as indicated by the CarbaResist kit. No further characterization beyond the CarbResist Kit was performed, and all 19 isolates were included in the subsequent analyses as carbapenemase-negative isolates.

Notably, carbapenem-resistant isolates that lacked a carbapenemase-based resistance mechanism were more frequently identified in the pre-COVID years than during the pandemic period. In 2018 and 2019, 50% (10/20) and 41.1% (7/17) of isolates, respectively, exhibited non-carbapenemase-mediated resistance. By contrast, in the subsequent three years (2020–2022), only two additional isolates with such resistance profiles were detected, accounting for less than 10% of the isolates in each of those years (Figure 1).

Overall, the diversity of carbapenemase genes was relatively low. Only eight different carbapenemase types (the five *bla*_GES_ are simply classified here as belonging to the same type), belonging to five distinct families, were identified.

**Figure 2:**
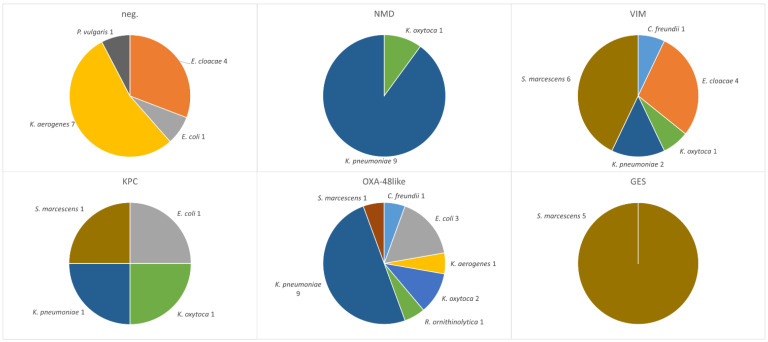
Detected carbapenemases (grouped by carbapenemase protein families): Species distribution of individual carbapenemases across all identified CRE isolates. A total of 55 carbapenemase genes were identified in 44 out of 63 isolates. The number next to each species name indicates the number of isolates. “neg” refers to 19 isolates that did not show a positive signal for any of the tested genes. Isolates that carried two or three carbapenemase genes simultaneously are shown separately for each carbapenemase.

Metallo-beta-lactamases accounted for the majority of the detected carbapenemases, comprising 45.5% (25 out of 55) of the total. VIM-1 producers (found in 15 isolates) and NDM-1 (in 10 isolates) were detected in nearly every year, with the exception of 2019, during which no NDM-1 was observed. OXA-48 producers (including 12 OXA-48, 4 OXA-181, and 2 OXA-244) were found in all years, accounting for 32.7% of all carbapenemases. Class A beta-lactamases were the least common and were represented by the KPC and GES family, but they were still detected in all years. In total, the gene encoding KPC-2 was found six times, and KPC-3 was detected once, and GES five times (Figure 1 and Figure 2).

Additionally, six isolates carried two or three carbapenemase genes simultaneously. The first of these, identified in 2019, was a *S. marcescens* carrying both VIM-1 and GES genes, in total four *S. marcescens* isolates revealed that pattern. One *S. marcescens* also harbors one for KPC-3. The remaining isolates harbored genes encoding for NDM-1 and OXA-48. Of these, four were *K. pneumoniae* (MLST ST11), with three isolated in 2021 and one in 2022. The final isolate was a *K. oxytoca* from 2022.

### 3.2. MLST

Multilocus sequence typing (MLST) was performed for all species with an available MLST scheme. The primary aim was to assess the genetic diversity of carbapenem-resistant isolates within each species and to identify potential dominant or nosocomial transmitted clones (Supplementary Table S1).

For the most common species, *K. pneumoniae*, the lowest diversity was observed: the 20 isolates exhibited 11 different MLST types. The predominant type was sequence type ST11, detected in four isolates. All ST11 isolates harbored both the NDM-1 and OXA-48 carbapenemase genes; three were isolated in 2021 and one in 2022. ST45 and ST437 were each identified in three isolates, all in the pre-COVID years 2018 and 2019. The two ST45 isolates from 2018 carried the NDM-1 gene, whereas the 2019 isolate was the only carbapenem-resistant *K. pneumoniae* isolate in this study lacking any of the examined carbapenemase genes. Similarly, the ST437 isolates from 2019 carried the same gene (encoding OXA-48), whereas the 2018 isolate differed by carrying the gene for VIM-1. ST101 was isolated twice in 2019, both instances carrying the gene for KPC-2. In contrast, the two ST147 isolates differed both in year of isolation (2018 and 2020) and carbapenemase gene: KPC-2 in 2018 and NDM-1 in 2020. All remaining ST types were identified in single isolates only (Supplementary Table S1).

The 11 *E. cloacae* isolates exhibited eight distinct MLST types. ST66 was the most common, with three isolates, all carrying the gene for VIM-1. This type was detected once in 2018 and twice in 2020. The two ST730 isolates were found in different years and exhibited different resistance mechanisms. One isolate from 2018 expressed KPC-2, while the isolate from 2019 did not carry any carbapenemase. All other ST types were found only once (Supplementary Table S1).

Among the six *E. coli* isolates, only one MLST type, ST38, was detected more than once. Both ST38 isolates were collected in 2019, one carrying the OXA-48 gene and the other for OXA-244. Among the *K. oxytoca* and *C. freundii* isolates, no MLST type was observed more than once (Supplementary Table S1).

### 3.3. Antibiotic Resistance

**Figure 3:**
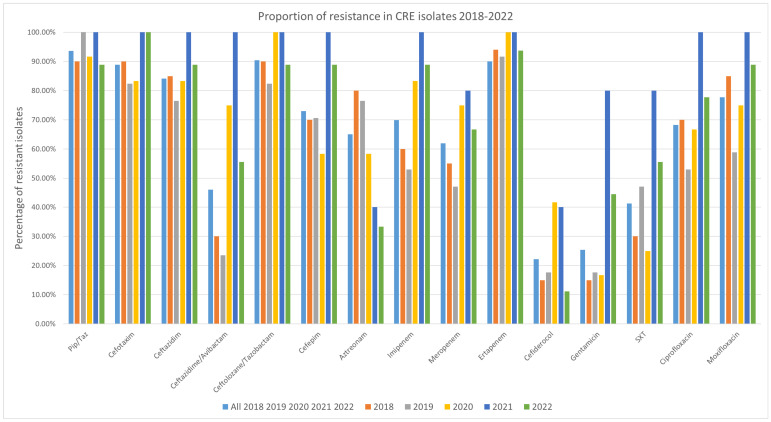
(Co)-Resistance in the CRE isolates. The first column (light blue) shows the proportion across all isolates (2018–2022). Number of isolates per year were 20 in 2018, 17 in 2019, 12 in 2020, five in 2021 and nine in 2022, in total 63 isolates. Abbreviations: Pip-Taz: piperacillin/tazobactam, SXT: sulfamethoxazole-trimethoprim.

Resistance to all tested antibiotics was detected among the isolates, although each antibiotic also had isolates for which it remained effective (Figure 3). Over the study period, the highest resistance rate among the tested antibiotics was observed for ertapenem and piperacillin/tazobactam, with 93.75% (59/63 isolates) exhibiting resistance. The isolates were most susceptible to cefiderocol, with 22.2% (14/63 isolates) revealing resistance (Figure 1 and Supplementary Table S1). 95% confidence intervals for all proportions were calculated using the Wilson method and are provided in the Supplementary Table S2.

When comparing resistance before and after the COVID-19 period, most of the tested antibiotics showed no significant changes. However, for cefazidime/avibactam, there was a noticeable increase in resistance starting from the first COVID-19 year, 2020. Resistance rose from 30% in 2018 and 23.5% in 2019 to 75% in 2020 and 100% in 2021, before slightly decreasing to 55.6% in 2022. This increase is linked to the rise in MBL-positive isolates, as all but one isolate (a *K. pneumoniae* carrying the *bla*_VIM-1_ gene) were resistant to ceftazidime/avibactam. However, five isolates were also found resistant and did not have genes for MBL.

In detail: In 2018, six isolates were resistant to ceftazidime/avibactam, three carrying *bla*_VIM-1_, two *bla*_NDM-1_ and one *bla*_OXA-48_. In 2019, four resistant isolates were detected, including one with *bla*_VIM-1_ and *bla*_GES_, while three isolates did not carry any carbapenemase gene. The highest number of resistant isolates was observed in 2020, with nine in total: six carried *bla*_VIM-1_ (one in combination with *bla*_GES_ and one with *bla*_KPC-3_ and *bla*_GES_), two carried *bla*_NDM-1_ combined and one *bla*_OXA-181_. In both 2021 and 2022, five isolates were resistant to ceftazidime/avibactam. Of these, three in 2021 and two in 2022 carried *bla*_NDM-1_ plus *bla*_OXA-48_, while two and three isolates, respectively, carried *bla*_VIM-1_ (one per year in combination with *bla*_GES_). It should be noted that no synergy testing of aztreonam and ceftazidime-avibactam was performed in this study, as some multidrug-resistant Gram-negative bacteria producing MBLs along with other β-lactamases.

For gentamicin, there was an increase in resistance during the second year of the COVID-19 pandemic (the first full COVID year). While 15% of isolates were resistant in 2018, 17.6% in 2019, and 16.7% in 2020, the proportion of gentamicin-resistant isolates rose to 80.0% in 2021, before dropping to 44.4% in 2022.

## 4. Discussion

Carbapenem-resistant Enterobacterales (CRE) remain a significant global public health concern. In low-endemicity countries such as Austria, the majority of cases appear to be travel-associated and occur as sporadic importations. This study investigated the impact of the COVID-19 pandemic on both the occurrence and the genetic background of CRE. This study is limited by a relatively small number of isolates (n = 63), which reduces the statistical power for detailed year-to-year comparisons. This is also attributable to the overall situation concerning CRE in Austria. In general, carbapenem-resistant isolates in Enterobacterales have remained relatively rare over the past years [10,11].

Nevertheless, the data demonstrate a clear and statistically significant decline in CRE prevalence during the COVID-19 pandemic in Southeastern Austria. The observed changes in certain species and resistance mechanisms during the pandemic may reflect the impact of external factors—such as global mobility, which was substantially reduced due to COVID-19-related travel restrictions—on resistance dynamics, particularly in low-endemicity countries.

Continued genomic surveillance, early detection of high-risk clones, and sustained antimicrobial stewardship efforts will be critical to preventing the re-emergence of CRE transmission chains in the post-pandemic period.

When examining the carbapenemases themselves, no marked differences were observed between the pre-pandemic and pandemic periods. However, notable changes emerged among isolates lacking carbapenemases. In 2018 and 2019, these isolates represented one of the most common categories of carbapenem-resistant Enterobacterales. From 2020 onward, however, they were rarely detected, with no more than one such isolate identified per year.

This trend is largely attributable to species shifts: *E. cloacae* and *K. aerogenes*, which frequently lacked carbapenemases, declined substantially during the pandemic.

Conversely, data from the European Antimicrobial Resistance Surveillance Network (EARS-Net) indicate a rising trend in invasive carbapenem-resistant *Klebsiella* spp. and *E. coli* across several European countries. In Germany, the proportion of carbapenem-resistant *K. pneumoniae* increased from 0.27% in 2019 to 0.47% in 2023. In Greece, the prevalence rose from 13% to 21% over the same period, while in Italy, carbapenem-resistant *E. coli* increased from 0.26% in 2019 to 0.31% in 2023. https://www.ecdc.europa.eu/sites/default/files/documents/Country_profiles_2023_2024 (accessed on 1 December 2024).

Notably, this study reports for the first time in Austria the emergence of isolates carrying two carbapenemases, such as NDM-1 and OXA-48. These dual-carbapenemase producers were entirely absent before 2020 and appeared for the first time during the initial year of the COVID-19 pandemic, subsequently increasing in frequency over the following years Most of these isolates belonged to *K. pneumoniae* sequence type ST11.

Although their emergence coincided with the pandemic, it is more plausibly explained by broader regional and international epidemiological trends. Similar isolates had already become relatively common in Austria’s neighboring countries prior to 2020. Therefore, the pandemic may have delayed—but likely did not cause—their introduction and spread in Austria [14,19,20].

Our MLST findings reveal high genomic diversity among CRE isolates, potential nosocomial transmission appears unlikely, though suspected cases would require higher-resolution typing. While *K. pneumoniae* ST11 clones (with dual carbapenemases) recurred during the pandemic, most other sequence types appeared sporadically and were likely introduced from external reservoirs. This reflects European-wide genomic surveillance showing CRE spread largely driven by horizontal acquisition rather than hospital outbreaks. At most, the COVID-19 pandemic may have slightly delayed their appearance in Austria [5,14,21,22,23].

Resistance trends for the key therapeutics—ertapenem or piperacillin/tazobactam, and gentamicin—remained high, as expected in CRE, affirming limited therapeutic options outside of cefiderocol, which retained the least resistance (~22%). Rates of resistance to cefazidime-avibactam and gentamicin surged during the pandemic, peaking in 2021. The sharp increase in ceftazidime–avibactam resistance reported in a Romanian study—from 28.1% in 2019 to 53.2% in 2021 (*p* < 0.00001)—corresponds with the trend observed in this study, where resistance rose from 23.5% in 2019 to 100% in 2021. These increases may reflect shifts in antimicrobial prescribing practices during the COVID-pandemic [24,25].

In contrast to other Western European countries, the Austrian dataset revealed a balanced carbapenemase distribution, although metallo-β-lactamases (MBLs) were slightly more frequent than other types. This contrasts with the CARBA-MAP study from Spain (2014–2018), where bla_OXA-48like_ was clearly dominant, followed by *bla*_VIM_ and *bla*_KPC_. A similar predominance of OXA-48 like enzymes was observed in the Netherlands between 2017 and 2019, though NDM producers also had a notable presence. Norway, a low-prevalence country like Austria, reported only sporadic detections of NDM, OXA-48 and VIM producers between 2015 and 2021 [26,27,28].

Greater heterogeneity is observed in Eastern and Southeastern Europe. According to a recent systematic review by Stefaniak and co-workers NDM dominates in Romania and Serbia, while KPC is the most frequent carbapenemase in Greece, and OXA-48 in Turkey. Studies focusing on *K. pneumoniae* lineages have revealed distinct regional distributions, such as blaOXA-48 ST101 in Serbia and Romania, *bla*_NDM_ ST11 in Greece, and *bla*_OXA-48-like_ ST14 in Türkey. In our study, ST11 was detected four times and carried both genes for NDM-1 and OXA-48. ST101 was also present but harbored the gene for KPC-2 instead, while ST14 was not found among our isolates [5,29,30,31,32].

Interestingly, the distribution of carbapenemases in Romania most closely resembles that observed in Austria. In Romania, metallo-β-lactamases accounted for 42.7% of all carbapenemases (38.4% NDM and 6.3% VIM), a proportion roughly equal to that of OXA-48-like enzymes at 43%. This is particularly notable given that Romania, unlike Austria or other Western European countries, is considered a high-prevalence country for carbapenem resistance. Overall, it must be taken into account that differences in study design, study period, and geographic focus—especially in countries with only a few available studies—can lead to data distortion or limited comparability [5,32,33].

It is also worth noting that many European surveillance studies have focused exclusively on carbapenemase-producing Enterobacterales (CPE), either by design or due to limitations of selective media or targeted PCR methods. As a result, non-carbapenemase-producing CRE are frequently underreported or entirely missed. Supporting this observation, a U.S. study from South Texas found that non-CPE were in fact more prevalent than CPE, particularly among *E. cloacae* and *K. aerogenes*, with resistance primarily driven by porin loss and β-lactamase overexpression rather than acquired carbapenemases. Similarly, a European surveillance study from 2016–2019 reported a substantial proportion of *K. pneumoniae* isolates lacking carbapenemases, alongside a notable frequency of K. aerogenes among non-CPE, confirming that non-enzymatic mechanisms contribute significantly to carbapenem resistance across species and regions [34,35].

The influence of labor migration and general travel movements on the CRE situation in Austria is supported by the fact that the composition of carbapenemases closely resembles that observed in Romania. Notably, Romanian nationals represented the largest group of labor migrants in Styria in 2019. They were followed by Germans, who originate from a region with low CRE prevalence, and citizens from Bosnia and Herzegovina, for which unfortunately only limited prevalence data are available. But this data show in contrast to Austria and Romania high dominance of OXA-48like [5,23,36].

This study has several limitations. Austria is a low-prevalence country for CRE, resulting in a relatively small sample size. All data originated from a single region in southeastern Austria, and regional effects cannot be fully excluded. The Infection Prevention and Control (IPC) screening program remained unchanged during the COVID-19 period; however, as not all patients in Austria are routinely screened, under-detection is possible. Moreover, due to pandemic-related restrictions, international travel activity was minimal, and travel history was not collected. The lack of detailed epidemiological metadata further limits the generalizability of the findings.

## 5. Conclusions

Carbapenem-resistant Enterobacterales (CRE) remain a global public health concern, but in low-endemicity countries such as Austria, their prevalence is strongly shaped by international travel and migration. During the COVID-19 pandemic, CRE prevalence appeared to decline, coinciding with reduced mobility. This period also saw the disappearance of non-carbapenemase producers and the first emergence of dual-carbapenemase-producing *K. pneumoniae* ST11. These findings highlight the strong influence of external factors on CRE dynamics and underscore the need for sustained genomic surveillance and antimicrobial stewardship in the post-pandemic era.

## Figures and Tables

**Table 1:** Species of CRE Isolates collected between 2018–2022 at the Medical University of Graz, Austria.

	*K. pneumoniae*	*E. cloacae*	*K. aerogenes*	*E. coli*	*S. marcescens*	*K. oxytoca*	*C. freundii*	*P. mirabilis*	*P. vulgaris*	*R. ornithinolytica*	Total
2018	6	3	5	2	1	1	1	0	1	0	20
2019	6	3	3	3	1	0	0	0	0	1	17
2020	2	5	1	0	2	1	0	1	0	0	12
2021	4	0	0	0	1	0	0	0	0	0	5
2022	2	0	1	1	1	3	1	0	0	0	9
Total	20	11	10	6	6	5	2	1	1	1	63

## Data Availability

The data presented in this study are available on https://doi.org/10.5281/zenodo.17357334 (accessed on 15 October 2025) and https://doi.org/10.5281/zenodo.17348024 (accessed on 14 October 2025).

## Supplementary Materials

The following supporting information can be downloaded at: https://www.mdpi.com/article/10.3390/pathogens14111130/s1, Supplementary Figure S1. PCR products for *bla*_GES_ for Serratia marcescens isolates. Supplementary Table S1. List of all carbapenem-resistant Enterobacterales isolates. The information includes isolate number, species, antibiotic resistance pattern, and detected carbapenemase genes, as well as MLST results. The alleles of the MLST profiles are provided in separate sheets of the Excel file, grouped by species. Supplementary Table S2. Antimicrobial resistance rates of carbapenem-resistant isolates.
