# Supplementary material for: Diversity in Carbapenemases in Enterobacterales in Southeastern Austria Before and During the COVID-19 Pandemic
Source: Pathogens. 2025 Nov 6;14(11):1130. doi: 10.3390/pathogens14111130 (PMC12655357; doi:10.3390/pathogens14111130)
Supplement: Supplementary file 1 [file pathogens-14-01130-s001.zip › Supplementary Figure S1.pdf]

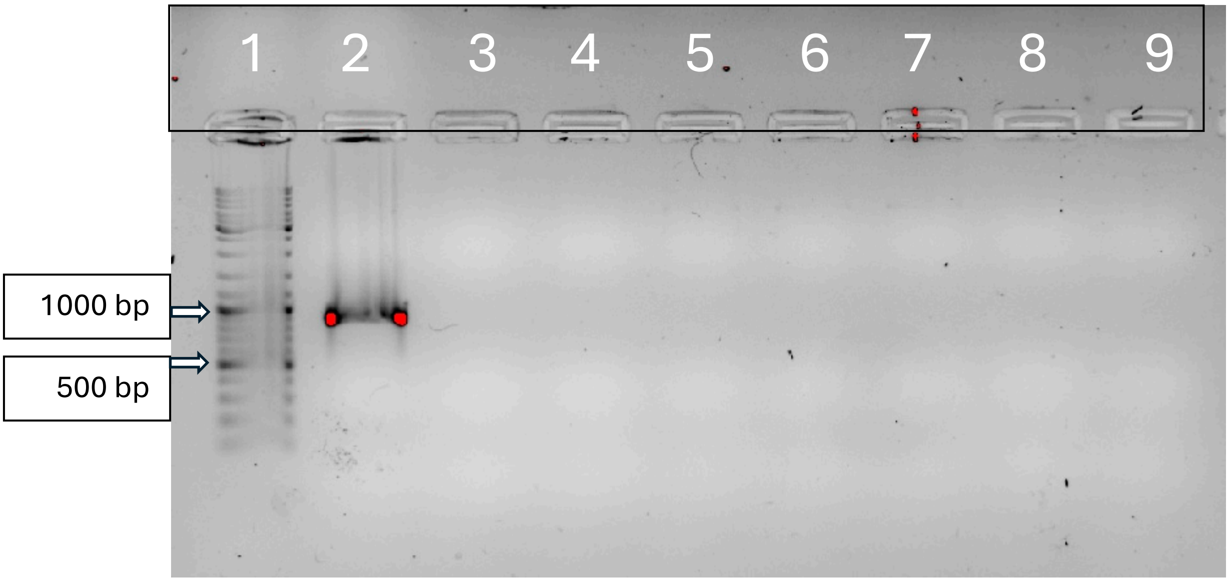

Supplementary Figure S1

PCR products for *bla*$_{GES}$ for Serratia marcescens isolates. Lane 1: 100 base pairs standard. Lane 2: Positive control *Klebsiella oxytoca* with *bla*$_{GES-1}$. Lane 3: S18/41. Lane 4: S19/19. Lane 5: S20/02. Lane 6: S20/118. Lane 7: S21/42. Lane 8: S22/01. Lane 9: negativ Control.
